# Recent Advances in Detection for Breast-Cancer-Derived Exosomes

**DOI:** 10.3390/molecules27196673

**Published:** 2022-10-07

**Authors:** Qin Tang, Xinying Xiao, Ranhao Li, Hailun He, Shanni Li, Changbei Ma

**Affiliations:** 1 School of Life Sciences, Central South University, Changsha 410013, China; 2 Xiangya School of Medicine, Central South University, Changsha 410078, China

**Keywords:** exosomes, breast cancer, nanomaterial, optical method, electrochemical method

## Abstract

Breast cancer is the most common malignant tumor in women, its incidence is secret, and more than half of the patients are diagnosed in the middle and advanced stages, so it is necessary to develop simple and efficient detection methods for breast cancer diagnosis to improve the survival rate and quality of life of breast cancer patients. Exosomes are extracellular vesicles secreted by all kinds of living cells, and play an important role in the occurrence and development of breast cancer and the formation of the tumor microenvironment. Exosomes, as biomarkers, are an important part of breast cancer fluid biopsy and have become ideal targets for the early diagnosis, curative effect evaluation, and clinical treatment of breast cancer. In this paper, several traditional exosome detection methods, including differential centrifugation and immunoaffinity capture, were summarized, focusing on the latest research progress in breast cancer exosome detection. It was summarized from the aspects of optics, electrochemistry, electrochemiluminescence and other aspects. This review is expected to provide valuable guidance for exosome detection of clinical breast cancer and the establishment of more reliable, efficient, simple and innovative methods for exosome detection of breast cancer in the future.

## 1. Introduction

### 1.1. Breast Cancer

Breast cancer is the most common malignant tumor in women, which is a serious threat to women’s health and life. According to the latest global cancer burden forecast data released by the World Health Organization’s International Agency for Research on Cancer (IARC) in 2020, breast cancer has become the most common malignant tumor in the world, accounting for 11.7% of new malignant tumor cases [1]. Among the malignant tumors of Chinese women, the incidence of breast cancer ranks first. There were 3.68 × 10^5^ new cases of breast cancer and 9.8 × 10^4^ deaths in China in 2018, which is one of the main causes of female death caused by tumors [2,3].

The exact pathogenesis of breast cancer is not clear, the related high-risk factors are difficult to control, and the primary etiological prevention is difficult to achieve, so the current prevention and control of breast cancer is mainly secondary prevention of "early detection, early diagnosis and early treatment". At present, the diagnosis of breast cancer is based on several aspects, including medical history, clinical physical examination, imaging examination, and histopathological diagnosis. Early detection and early diagnosis are very important to improve the curative effect and prognosis of breast cancer. Studies have shown that the stage of breast cancer diagnosis is an important factor affecting the prognosis of breast cancer [4,5]. The 5-year survival rate of patients with clinical stage I breast cancer was 93%~99.7%, 85%~96.4% for clinical stage II patients, 48%~83.1% for clinical stage III patients, and 14%~41.8% for stage IV patients [6,7,8,9]. The 5-year survival rate of patients with clinical stage I–II breast cancer is about 50% higher than that of patients with clinical stage III–IV breast cancer. It is obvious that early detection of breast cancer is an important way to reduce the mortality of breast cancer. However, because most of the patients with early breast cancer do not have any clinical symptoms, more than half of the patients are in the middle and advanced clinical stage at the time of diagnosis, which seriously affects the quality of life of patients with breast cancer and reduces the cure rate and survival rate of breast cancer. Therefore, it is urgent to develop a more simple and efficient method for breast cancer diagnosis.

### 1.2. Exosomes

Exosomes are extracellular vesicles (EVs) with a diameter of 40–160 nm (average 100 nm), which can be produced by all kinds of cells throughout the body [10,11]. In the process of exosome biogenesis, the cell membrane firstly sinks inward to form multivesicular bodies (MVBs) and intersects with other vesicles and organelles in the cell to obtain exosome components. MVBs fuse with the plasma membrane to release the exosome into the extracellular space and be absorbed by adjacent or distant cells to play the role of cellular communication [10,12]. Because exosomes can be derived from all cells, they have diversity and heterogeneity. Exosome components include DNA, RNA, lipids, cytoplasm, metabolites, and cell-surface proteins. Their heterogeneity can reflect the size and content of exosomes, the effects on receptor cells, and the characteristics of origin cells [10,13].

In recent years, the function of the exosome has been continuously explored and has already been used in disease diagnosis and treatment. Exosomes are associated with immune response, viral pathogenicity, cardiovascular disease, central nervous system disease, and cancer progression [10]. For example, activated T cells can recruit exosomes derived from dendritic cells containing MHC-II and down-regulate immune response [14]; cells infected with hepatitis virus can release exosomes containing viral nucleic acid or protein components, participating in virus transport and immune escape to improve its pathogenicity [15]; platelet-derived exosomes can reduce the expression of CD36 in macrophages, thus reducing ox-LDL uptake and preventing atherosclerosis [16]. MiRNAs are the most commonly used tissue-specific exosome component. They can be used as biomarkers for disease diagnosis, which are mainly applied in cancer, e.g., miR-638 can be used as a diagnostic marker of colorectal cancer [17]. In the aspect of disease treatment, exosomes have a strong ability to relocate to target cells. They can penetrate the biological barrier, making them promising targeted drug carriers. By loading with the exosomes derived from activated M1 macrophages, paclitaxel can reduce its toxicity and improve bioavailability [18].

### 1.3. Exosomes and Breast Cancer

Exosomes play an important role in the occurrence and development of breast cancer, tumor microenvironment formation, angiogenesis, invasion, metastasis and recurrence, and drug resistance [10,19,20]. On the one hand, breast cancer cells can release exosomes into the tumor microenvironment and act on cancer-associated fibroblast (CAF), cancer stem cells, and other stromal cells in the breast cancer microenvironment, affecting intercellular and intracellular signal transduction, promoting cancer cell survival and proliferation, invasion and migration, metabolic recombination, and tumor angiogenesis. On the other hand, other stromal cells in the breast cancer microenvironment can also secrete exosomes, transmit material information through exosomes, and regulate the occurrence and development of breast cancer [10]. For example, miR-105 in the exosomes secreted by breast cancer cells can promote the proliferation and survival of cancer cells, promote tumor growth, inhibit the connection between vascular endothelial cells, and help cancer cells exudate blood vessels and metastasize to distant places [21,22]. The exosomes secreted by CAF containing a large amount of growth factors can promote the growth and proliferation of cancer cells [23], while the CD81 positive exosomes secreted by CAF can promote the invasion and migration of breast cancer cells through the Wnt signal pathway [24]. 

With the more in-depth study of the role of exosomes in breast cancer, as a potential target for early diagnosis, curative effect evaluation, and clinical treatment of breast cancer, the important value of exosomes in the diagnosis and treatment of breast cancer has been explored more and more. Tumor-derived exosomes can be isolated from patients’ ascites fluid, urine, and pleural effusions. Assessment of breast-cancer-derived exosomes can be used to develop non-invasive diagnostic methods that will help to monitor disease progression, therapeutic efficacy, and resistance mechanisms, and also help to judge the tumor status (stage and subtype) [25,26]. For instance, breast milk exosomes with higher levels of TGFβ2 are positively correlated with higher breast cancer risk [27,28]. In the patients with breast cancer, the circulating exosomal miRNA-21 and miRNA-1246 were abundant [29], and in patients with triple-negative breast cancer, circulating exosomal miRNA-373 was noticeably elevated [30]. In addition, ADAM10, a metalloprotease, CD9, Annexin-1, and HSP70 were specifically at a high level in exosomes obtained from pleural effusion/sera of patients with breast cancer [31]. Therefore, the researchers concluded that circulating exosomal miRNA profiles and exosomal proteins may be used as a potential diagnostic biomarker for breast cancer. By investigating exosome-associated biomarkers in breast cancer patients undergoing chemotherapy, Wang et al. found that the circulating exosomes carrying TRPC5 were significantly correlated with the expression level of TRPC5 in breast cancer tissues and response to chemotherapy. Additionally, increased circulating exosomes carrying TRPC5 after chemotherapy preceded cancer progression and predicted acquired chemotherapy resistance. Therefore, the detection of TRPC5-positive exosomes can be used to monitor chemotherapy resistance in real time [32]. Other researchers found that the level of serum exosomal lncRNA HOTAIR from breast cancer patients was significantly higher and it significantly decreased in all patients 3 months after the operation, suggesting that a high expression level of serum exosomal HOTAIR is related to tumor burden and disease invasiveness and it was also found to be associated with a poorer response to neoadjuvant chemotherapy and TAM therapy [33]. Studies found that some miRNAs, such as miR-1246, miRNA-19a, miRNA-340-5p, and miRNA-93-5p, etc. are related to breast cancer progression, metastasis, and recurrence, which indicated that these exosomal miRNAs may be used as a potential biomarker of prognosis for breast cancer [34,35]. The clinical application of exosomes in breast cancer has broad prospects, while accurate and sensitive detection of breast cancer specific exosomes is the premise to realize these significant values.

## 2. Common Methods of Exosomes Separation

In order to quantify biological samples such as cell culture medium and exosomes in body fluid, the exosomes in the sample should be separated and enriched first [36]. In the sample, in addition to the exosomes, there are usually many cell fragments and other kinds of membrane vesicles [37]. At present, the commonly used separation methods are ultracentrifugation, ultrafiltration, precipitation, and exosomes separation based on immune affinity [38]. In this paper, some commonly used exosomes separation methods are briefly summarized (Table 1).

## 3. Detection of Breast-Cancer-Derived Exosomes

In the past research on exosome detection, researchers focused on optimizing several traditional detection methods, such as western blotting [50], tracking and analysis of nanoparticles (NTA) [51], flow cytometry [52] and enzyme-linked immunosorbent assay (ELISA) [53], etc. However, the abundance of tumor exosomes in biological fluids is very low, and their signals are easily hidden by heterogeneous exosomes [54]. Moreover, the traditional detection methods have some disadvantages, such as a large amount of samples, expensive instruments, low sensitivity, and so on. Even if there are optimization measures such as signal amplification technology, they essentially require complex sequence design, fine surface modification, and tedious sample preparation steps [55]. In recent years, a number of emerging breast cancer exosome detection methods have emerged, such as colorimetry, fluorescence, surface-enhanced Raman scattering, and other optical methods [56], as well as some electrochemical, thermal signal, and other related methods. They generally have better sensitivity and higher stability, and there is experimental and clinical evidence to confirm their application prospects, which we review below. 

### 3.1. Optical Method

#### 3.1.1. Colorimetric Method

The colorimetric method can determine whether a substance exists and the concentration of the substance by comparing the changes in the color or color depth of the solution. The colorimetric method can be used to detect exosomes intuitively, reduce the demand for instruments, simplify the processing process, and be suitable for timely detection [57]. Horseradish peroxidase (HRP), as a common peroxidase, has high substrate specificity and catalytic activity, and is a common substrate in traditional colorimetry. Although natural enzymes have many advantages, their disadvantages still limit their wide application, such as complex preparation, purification and storage, and structural instability. In order to overcome the limitations of HRP, many studies, which improve the colorimetric method on the basis of HRP, have been reported in recent years, which develop the detection technology of breast cancer exosomes.

##### General Material

**Figure 1:**
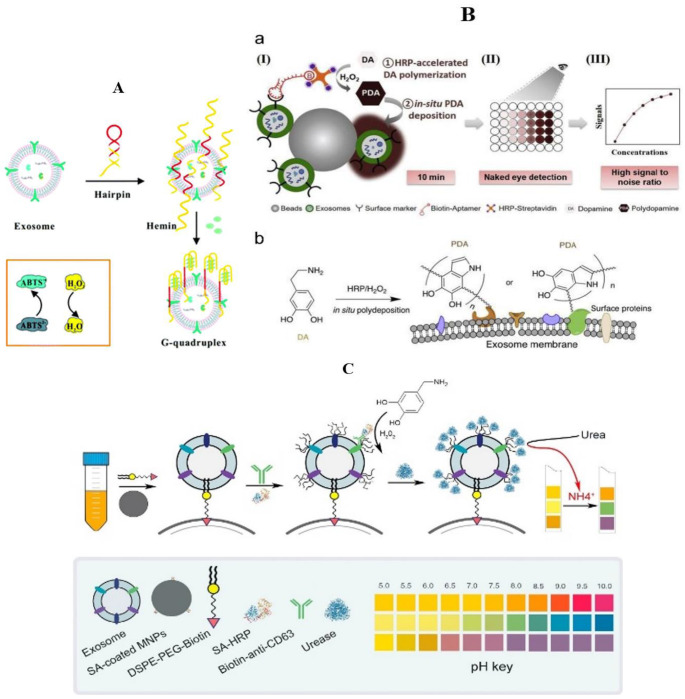
Detection of exosomes in breast cancer by the colorimetric method (general material). (**A**) Schematic illustration of the assay for the detection of exosomes by binding to hairpin structures based on the combination of the MUC1 aptamer and G-quadruplex-mimetic enzyme [61]; (**B**) schematic illustration of the proposed aptasensor with HRP accelerated dopamine polymerization and deposition for exosome detection [57]; (**a**) (I) exosomes were captured by aptamer. (II) colorimetric detection. (III) absorbance signals can also be quantified. (**b**) scheme shows the poly-deposition process of PDA onto surface proteins of exosomes in situ. (**C**) schematic illustration for magnetic capture of exosomes, HRP-mediated PDA engineering of exosomes, and urease immobilization for point-of-care testing [62].

Hemin/G-quadruplex possesses horseradish peroxidase-mimicking catalytic activity, and can efficiently catalyze the H_2_O_2_-mediated oxidation of several substrates accompanied by obvious color change, which is superior to native peroxidases because of their high chemical and thermal stability, low cost, simple preparation, and easy modification [58,59,60]. Yu et al. designed a sensitive, simple, and low-cost colorimetric aptamer sensor that combines highly specific mucin 1 (MUC1) aptamers with heme/G-quadruplex for the detection of breast cancer exosomes (Figure 1A) [61]. They chose MUC1, which is overexpressed in MCF-7 cells, as an ideal target molecule for the detection of exosomes secreted by MCF-7 breast cells. When the hairpin-like aptasensor specifically bound to the MUC1 expressed on the surface of MCF-7 exosomes, the original DNA hairpin was opened and triggered the self-assembly of G-quadruplex subunits into a catalytically active G-quadruplex DNAzyme with the assistance of hemin. Subsequently, the reduction of H_2_O_2_ generated colorimetric signals, the blue color change caused by the catalytic oxidation of ABTS could be easily observed by naked eyes, and the detection results could be read out directly through a microplate reader. This method reduces the exosome detection limits to 3.94 × 10^5^ particles/μL and exhibits the advantages of high sensitivity, convenience, time-saving, fewer instruments, and easy observation by the naked eyes.

In order to further realize the high sensitivity of exosome detection, Xu et al. captured target exosomes with latex beads via aldimine condensation, followed by bio-recognition using a specific CD63 aptamer, which was conjugated to horseradish peroxidase (HRP) through biotin–streptavidin binding (Figure 1B) [57]. Colorimetric detection was achieved in 10 min via enzymatic catalysis to produce dark-colored polydopamine (PDA) from colorless substrate dopamine (DA) in an especially prepared H_2_O_2_ reaction solution, which could be directly observed by the naked eye. Signal quantification was carried out by absorbance measurement. The color intensity correlates to the CD63 amount and the limit of detection can be as low as 7.7 × 10^3^ particle/mL, improved by 3–5 orders of magnitude from conventional dot-blot methods. This aptasensor showed specificity to HER2 and integrin αvβ6 positive, cell culture-derived, and breast and pancreatic cancer-derived exosomes, respectively.

What is refreshing is that the low-cost, widely used, and commercially available pH test paper has also been applied in the detection of exosomes. Via HRP-mediated promotion of mussel-inspired surface engineering and reagent-free functionalization of urease molecules, a novel approach for exosome quantification using pH test paper has been developed (Figure 1C) [62]. Different from the traditional colorimetric sensors, they use HRP to exert their activity to rapidly catalyze the formation of PDA film on exosome surfaces, and then take advantage of the remarkable reactivity of PDA with proteins to hydrolyze urea into ammonia and carbon dioxide to raise the pH value of the solution. By establishing the relationship between exosome recognition and the change of pH value of the sensing solution, we can employ the l pH test paper to quantitatively analyze exosomes. The pH-responsive bioassay enables sensitive detection of exosomes with a detection of limit down to 4.46 × 10^3^ particles/µL and can be successfully applied for the determination of exosomes in clinical specimens.

##### Nanometer Material

A variety of nanomaterials, such as the nanoparticles of Fe_3_O_4_ [63,64], gold [65], cerium oxide [66], carbon-based nanotubes [67], or graphene oxide nanosheets [68], have been observed to possess unique catalytic activities that mimic natural enzymes, termed nanozymes [69]. Compared with biological enzymes, nanozymes are more stable, less expensive, and easier to store, with a few showing higher catalytic activity.

**Figure 2:**
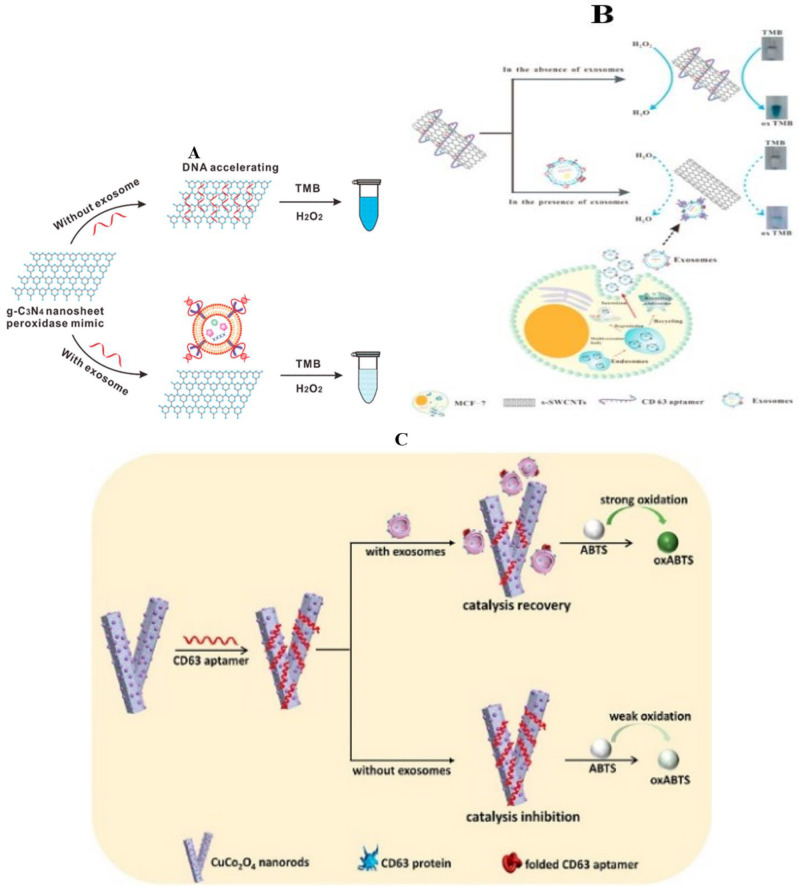

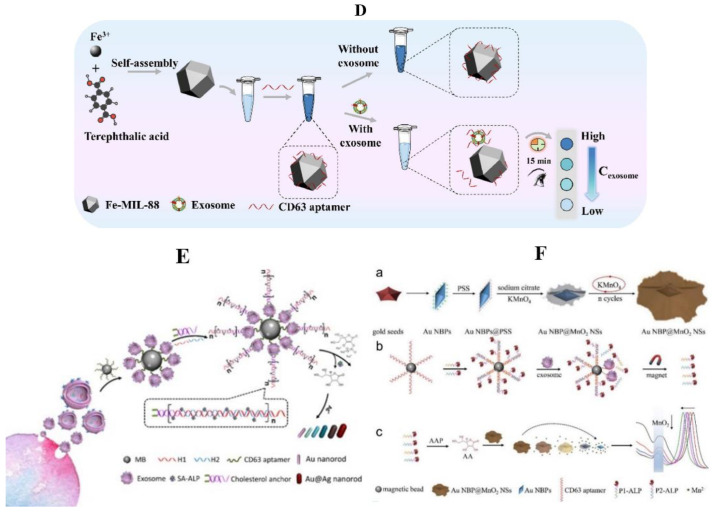
Detection of exosomes in breast cancer by the colorimetric method (nanomaterials). (**A**) illustration of DNA aptamer accelerating the intrinsic peroxidase-like activity of g–C_3_N_4_ NSs for the detection of exosomes [73]; (**B**) schematic representation of the detection mechanism of the proposed method for exosomes [74]; (**C**) schematic representation of the mechanism for label-free detection of exosomes based on CD63 aptamer inhibiting oxidase activity of CuCo_2_O_4_ nanorods [75]; (**D**) illustration of CD63-aptamer-functionalized Fe–MIL-88 for the detection of exosomes [79]; (**E**) schematic illustration of the mechanism for multicolor visual detection of exosomes based on HCR and enzyme-catalyzed metallization of AuNRs [81]; (**F**) (a) schematic of the fabrication process of Au–NBP@MnO_2_ NSs, (b–c) schematic illustration of the plasmonic colorimetry for exosome detection via competitive reaction and etching of Au–NBP@MnO_2_ NSs [85].

Graphitic carbon nitride nanosheets (g-C_3_N_4_NSs) are a type of graphene-like, carbon-based, two-dimensional (2D) nanomaterial, which were found to possess intrinsic peroxidase-like activity and were employed for the detection of glucose in serum or uric acid in urine [70,71,72]. In order to obtain nano-enzymes with higher catalytic activity and improve the colorimetric device for exosome detection, the capability of single-stranded DNA (ssDNA) in enhancing the intrinsic peroxidase-like activity of the g-C_3_N_4_ nanosheets (NSs) plays a role (Figure 2A) [73]. The ssDNA aptamers for CD63 are adsorbed onto g-C_3_N_4_NSs and can enhance their intrinsic peroxidase activity, accelerating the oxidation of 3,3′,5,5′-tetramethylbenzidine (TMB) by H_2_O_2_ and generating a product with an intense blue color. The maximum reaction rate of the H_2_O_2_-mediated TMB oxidation catalyzed by the ssDNA-NSs hybrid was at least four times faster than that obtained with unmodified NSs. Additionally, measurement by visible absorbance at the product’s λmax can lead to absolute quantification of exosomes based on the amount of CD63. The sensor recognized the differential expression of CD63 between the exosomes produced by a breast cancer cell line (MCF-7) and a control cell line (MCF-10A). Moreover, a similar trend was detected in the circulating exosomes isolated from the sera samples collected from breast cancer patients and healthy controls.

Tubular nanomaterial was considered an ideal candidate for nanodevice applications over the past several decades, which can catalyze TMB-H_2_O_2_ with high catalytic activity; they were needed for treatment by sulfuric and nitric acid, which was time-consuming, sophisticated, and even destroyed the integrity of single-wall carbon nanotubes (SWCNTs). Besides, the SWCNTs were had water solubility, which made the result of detection not accurate enough. In order to overcome this series of problems, Xia et al. prepared s-SWCNTs that are rich in carboxyl groups and have good water solubility, and designed a visible and colorimetric aptasensor based on DNA-capped single-walled carbon nanotubes for the detection of exosomes (Figure 2B). Aptamers, specific to exosome transmembrane protein CD63, are absorbed onto the surface of s-SWCNTs and improve the minic peroxidase activity of s-SWCNTs, which can efficiently catalyze H_2_O_2_-mediated oxidation of TMB and lead to a change from colorless to blue in solution [74]. After adding exosomes, the aptamers are bound with CD63, leaving from the surface of s-SWCNTs through conformational changes, which results in the color of the solution changing from deep to moderate, and this can be observed by the naked eye and monitored by UV–vis spectrometry. Under optimal conditions, the detection of limit (LOD) is 5.2 × 10^5^ particles/μL. Using this method, it is found that this aptasensor quantification results in an approximately 1.5-fold increase in the presence of exosomes in the breast cancer patients’ serum compared with healthy people. 

Metal nanometer materials have also been widely used in the detection of exosomes of breast cancer. A label-free exosome detection method based on the target-responsive controllability of the oxidase-like activity of Cu/Co bimetallic metal–organic frameworks (CuCo_2_O_4_ nanorods) has been proposed (Figure 2C) [75]. In the absence of exosomes, the oxidase-like activity was inhibited due to the adsorption of CD63 aptamer onto the nanorods’ surfaces. In the presence of exosomes, CD63 aptamer was disassembled from CuCo_2_O_4_ nanorods by virtue of CD63 aptamer-exosome recognition, which resulted in the recovery of oxidase-like activity. Accordingly, a sensitive colorimetric method for detecting exosomes was established with a detection limit of 4.5 × 10^3^ particles mL^−1^. The method was further applied in distinguishing healthy people and breast cancer patients by testing exosomes in the serum samples and showed satisfying differentiation ability.

MOFs are commonly accepted as a class of highly-ordered crystalline materials formed by the self-assembly of metal ions and organic linkers via coordination electrons, having unique properties such as highly exposed surfaces, abundant active sites, and excellent catalytic activity [76,77,78]. Fe–MOFs have recently emerged as promising enzyme mimics. By engineering DNA ligands on the surface of an iron-based metal–organic framework (Fe–MOF), a rapid, low-cost, and facile method for detecting exosomes has been developed (Figure 2D) [79]. Aptamers of exosomal transmembrane CD63 protein (CD63-aptamers) were utilized as both an optically active layer and an exosome-specific recognition element to engineer a Fe–MOF bio-interface for high-efficiency regulation of the catalytic behavior of Fe–MOF toward the chromogenic substrate. The specific binding of exosomes with CD63-aptamers altered the conformation of DNA ligands on the surface of Fe–MOF, contributing to sensitive variation in Fe–MOF catalytic activity. This directly produced a distinct color change and enabled the visual detection of exosomes. Via one-step ‘‘mixing-and-detection”, the Fe–MOF bio-interface exhibited excellent performance in quantitative analysis of exosomes derived from human breast cancer cell lines with a detection limit of 5.2 × 10^4^ particles/μL. the method was successfully applied to the identification of exosomes in serum samples

Compared with single-color change, lively color variation is more easily distinguished by naked eyes. Plasmonic noble metal materials are potential candidates. Compared with similarly sized gold nanoparticles, Au NRs have better performance due to an inherently higher sensitivity to the local dielectric environment, and more vivid color changes are presented when growth or etch of Au NRs occurs [76,80]. Accordingly, Zhang et al. presented a sensitive multicolor visual method for exosome detection based on enzyme-induced silver deposition on gold nanorods (AuNRs) (Figure 2E) [81]. Exosomes were captured by magnetic bead-labeled CD63 aptamer, and, then, cholesterol-modified DNA probes were spontaneously inserted into the exosomal lipid membrane. The ends of the DNA probes act as the initiators to trigger the HCR for signal amplification, leading to enhanced ALP loading and thus boosting the ascorbic acid generation. Silver ions were reduced by ascorbic acid, and silver shells were formed on AuNRs, giving rise to the blue shift of the longitudinal localized surface plasmon resonance peak. The concentration of exosomes can be obviously distinguished with naked eyes via the vivid color variation. The detection limit of this method is as low as 1.6 × 10^2^ particles/μL by UV–vis spectroscopy and 9 × 10^3^ particles/μL by naked eyes. The analysis of clinical samples by this method showed that there were significant differences in exosomes in serum samples from three breast cancer patients and two healthy volunteers. 

Sensitive multicolor visual detection of exosomes was realized for the first time with the help of AuNRs, whereas the detection procedure is tedious [81]. Gold nanobipyramids (Au–NBP) have two pentagonal pyramids with sharper tips at their apexes, which make them more sensitive than AuNRs to the refractive index [82,83,84]. To improve the performance of colorimetric substrates and simplify the experimental process, by using Au–NBP with remarkable optical properties, some researchers presented a highly sensitive plasmonic colorimetric biosensor for exosome quantification (Figure 2F) [85]. The sensing strategy mainly includes two steps: exosome-triggered competitive reaction and etching of Au–NBP@MnO_2_ nanosheet nanostructures. A competitive reaction between exosomes and placeholder chains induced by exosomes can translate the signal of exosomes into the amount of alkaline phosphatase, which simplifies the experimental process and amplifies the signal. The detection limit of this scheme was 1.35 × 10^2^ particles/μL, which is more sensitive than the previously reported colorimetric method. Using this method to analyze the exosomes in the serum of healthy people and breast cancer patients, the average concentration of exosomes from breast cancer patients was significantly higher (8.6-fold) than that from healthy volunteers. 

As a visual method for the detection of tumor exosomes, the colorimetry method also has the characteristics of convenience, sensitivity, and reliability, and is especially suitable for the detection of exosomes as a biomarker in the conventional clinical environment. However, as the technology and equipment of colorimetry are based on labeled samples, they depend on additional sample preparation steps, increasing the difficulty and overall related costs. For large-scale applications in exosome detection, colorimetry still has much room for improvement.

#### 3.1.2. Fluorescence Method

In the process of detecting exosomes by fluorescence, fluorescent molecules are used to label specific antibodies, DNA probes, and nucleic acid aptamers. These labeled antibodies, DNA probes, and aptamers can produce an antigen–antibody reaction or nucleic acid molecular hybridization with the exosomes to be measured. Under the irradiation of a specific-wavelength laser, the reaction mixture can display a fluorescence signal, and thus realize exosome detection by the imaging system. The operation of the fluorescence method is relatively simple, but the difficulty lies in designing suitable antibodies, probes, or aptamers, amplifying the fluorescence signal, reducing the lowest detection limit, and improving the sensitivity. Due to its simple operation and diversity, fluorescence is the most commonly used exosome detection technique at present.

##### General Material

The current fluorescence detection of exosomes mostly depends on nucleic acid aptamers [86]. Nucleic acid aptamer, also known as a chemical antibody, is a short DNA or RNA oligonucleotide fragment screened by Systematic Evolution of Ligands by EXponential enrichment (SELEX) according to the target molecular sequence. The aptamer can specifically bind to the target substance, whose effect is similar to antigen–antibody reaction. Compared with antibodies, aptamers have a wider range of target molecules, stronger affinity and specificity, simpler preparation methods, and more stable structures. Therefore, using aptamer as a fluorescence carrier to detect exosomes has gradually become a research hotspot.

**Figure 3:**
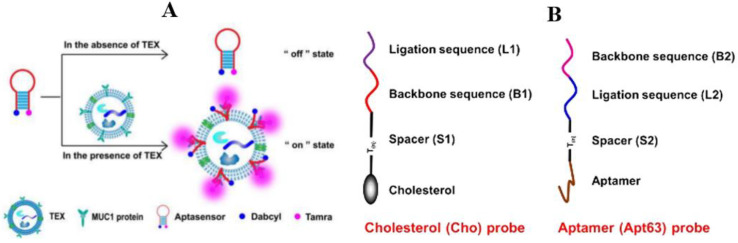

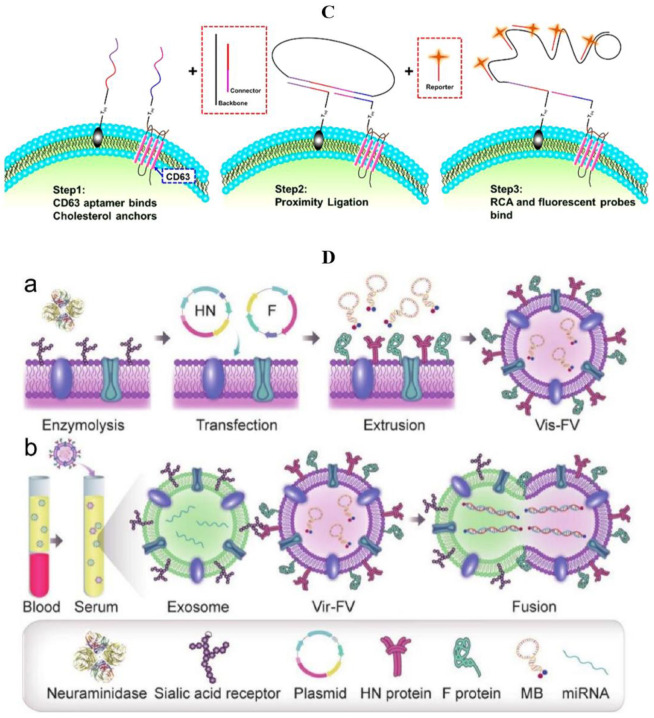
Detection of exosomes in breast cancer by the fluorescence method (nucleic acid aptamers). (**A**) Schematic illustration of the aptasensor for TEX detection [87]; (**B**) structure and functional domains of anti-CD63 aptamer probe and cholesterol probe [88]; (**C**) details of AcmPLA for target exosome recognition and signal amplification; CCS, cell culture supernatant [88]; (**D**) schematic illustration of the virus-mimicking fusogenic vesicles (Vir-FVs) for the rapid detection of exosomal miRNAs [92]: (a) preparation of the Vir-FVs. (b) The fusion between the Vir-FVs and exosomes caused by HN and F protein leads to the hybridization of the MB with the target miRNA.

Zhang et al. reported an “on–off” fluorescent aptamer sensor activated by the overexpression of MUC1 exosomes in breast cancer (Figure 3A) [87]. Firstly, they demonstrated that MUC1 was highly expressed on the exosome surface of MCF-7 breast cancer cells, which was significantly higher than in other cells. Based on the structure of MUC1, Zhang and his team then designed hairpin MUC1 aptamers and combined Tamra fluorescent probes and Dabcyl quenchants at each end of the aptamers. Under normal conditions, Tamra binds to Dabcyl, and the aptamer does not emit fluorescence. However, when MCF-7 exosomes appear in the environment, the aptamers are specifically coupled with the MUC1 on exosomes, which changes the hairpin structure, resulting in the separation of Tamra and Dabcyl. Tamra emits strong fluorescence, and the results can be read directly by a fluorescence spectrophotometer. By comparing the detection results of MCF-7 and Hs578Bst (normal breast cell), it was verified that the aptamer sensor has a specific recognition-switching performance in exosomes of breast cancer. In the serum of breast cancer patients, the value of the exosome fluorescence signal was also significantly higher than healthy controls. This method has a good dynamic range (1.0 × 10^5^~1.6 × 10^6^ particles/μL) for MCF-7 exosomes, and its detection limit is 4.2 × 10^4^ particles/μL. By the way, it takes only 32 minutes from adding an exosome to generating a signal in the detection process, which proves that this method is suitable for detecting exosomes. An aptamer-cholesterol-mediated proximity ligation assay (AcmPLA) is a method of using double probes to improve detection accuracy (Figure 3B,C). One aptamer probe is used to identify the intrinsic protein CD63 on the exosome’s surface, and the other targets the cholesterol in the lipid layer of exosomes vesicles. When two aptamers combine with the target substances, the Connector and Backbone carried by the probe can be connected due to their adjacent positions, which is called “Proximity Ligation”. The annular framework formed by ligation can amplify the signal by rolling circle amplification (RCA), which is a single probe that can bind to multiple fluorescent molecules to enhance the specific fluorescence signal [88]. Similarly, by detecting serum and urine samples of clinical patients, the enzyme-free amplification method of “Bivalent Cholesterol Anchor Triggered Target Conversion” (proposed by Wang et al.) was confirmed to be appropriate for all kinds of cancer exosome detection [89].

As mentioned above, exosome detection methods using aptamers as fluorescence carriers often have good versatility [90]. In the case of only changing the aptamers, the same fluorescence probe and signal amplification method can often be applied to the diagnosis of other tumor exosomes. For example, Liu and his team used a similar method to Zhang [87] and successfully detected the exosome of A549 non-small-cell lung cancer cells by targeting MUC-1 [91]. To sum up, the combination of aptamer and fluorescent probe for tumor exosome detection has the advantages of good versatility, convenient preparation, strong affinity and specificity, stable structure, and so on. There is no doubt that these aptamer-fluorescence detection methods have bright application prospects.

In addition, the fluorescence method can also be combined with the traditional exosome detection method through a specific carrier [92]. Gao et al. reported a new method for detecting EVs using virus-like fusion vesicles (Vir-FV) (Figure 3D). The fusion protein on Vir-Fvs can specifically target sialic-acid-containing receptors on EVs, inducing the fusion of Vir-Fvs and exosomes. When the contents of the two vesicles are mixed, the signal molecules wrapped in the VIR-FVs specifically hybridize with target miRNAs in exosomes to produce fluorescence. Finally, the signal was quantified by flow cytometry to complete the detection of EVs. In the experiment on MCF-7 cells, Gao et al. set the breast cancer biomarker miR-21 as the detection target, and successfully completed the EV identification, which proved its application prospect. From this perspective, by combining the fluorescence method with the traditional one, researchers can reduce costs and optimize the traditional method while also ensuring the detection effect [52].

Signal amplification methods are an important guarantee for the implementation of exosome detection [93]. By combining these methods with fluorescence, researchers can significantly increase the limit of detection (LOD), thus improving the sensitivity of exosome detection.

**Figure 4:**
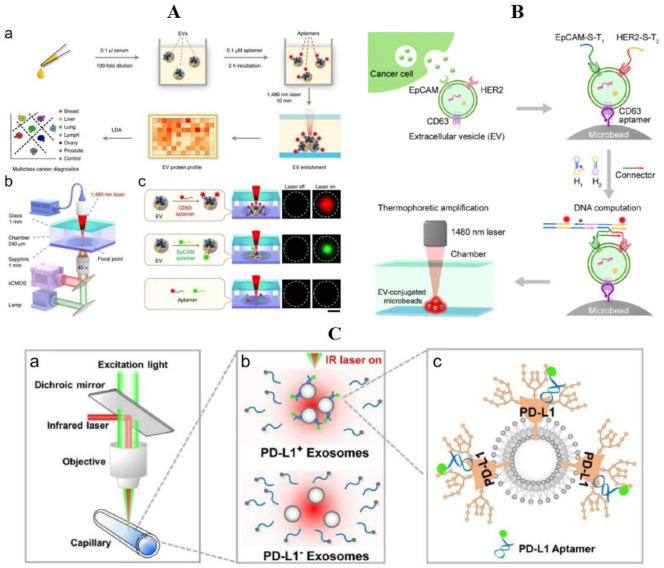
Thermal electrophoresis combines aptamers in the fluorescence method. (**A**) Overview of TAS for the profiling of surface proteins of EVs: (a) schematic of the TAS procedure. Using a panel of seven aptamers, the protein signatures of EVs in 0.1 μL human serum were profiled by TAS and used for multiclass cancer diagnostics. (b) Schematic of the setup of TAS. The 1480 nm infrared laser beam was focused onto the bottom of the microchamber for EV accumulation. sCMOS, scientific complementary metal oxide semiconductor. (c) Schematic of TAS for the enrichment and detection of aptamer-bound EVs. An amplified fluorescence signal was observed for aptamer-bound EVs after thermophoretic concentration. Owing to small sizes, free aptamers cannot be enriched. The working area is indicated by a dashed circle with a diameter of 100 μm. Scale bar, 50 μm [96]; (**B**) schematic of thermophoresis-mediated DNA computation on EV membranes [98]; (**C**) working principle of HOLMES-ExoPD-L1 analysis [99].

Thermal electrophoresis is often used as an enrichment technique in conjunction with fluorescence [94]. The phenomenon in which molecules move along the temperature gradient is called "thermal swimming". In the process of ion electrophoresis, the temperature gradient can often induce convective flow, resulting in the opposite direction of ion movement to the current, which is called "thermal electrophoresis". By precisely manipulating microns/nanoparticles on the temperature gradient, thermal electrophoresis can locally accumulate substances without labeling [95]. Because DNA has good thermal stability, DNA sensors can be combined with thermal electrophoresis technology. Thermophoretic aptasensor (TAS) uses a group of aptamers to label exosome proteins, accumulate EVs by size-dependent thermal electrophoresis, amplifiy the fluorescence signal produced by aptamer binding, and analyze exosome proteins sensitively to diagnose cancer [96]. Liu et al. designed a TAS that does not require the pre-separation of EVs and which analyzes the surface proteins of EVs in serum of cancer patients by thermal swimming enrichment and linear discriminant analysis (Figure 4A). The TAS’s working principle is that the aptamer-bound EVs are enriched by thermal electrophoresis in a size-dependent way to produce an amplified fluorescence signal, whose intensity indicates the expression level of EVs’ surface proteins. This method is simple, sensitive, and economical, and has been used to detect EVs markers to distinguish metastatic breast cancer (MBC) from non-metastatic breast cancer (NMBC) [97].

Li et al. used EpCAM and HER2 as a marker for detecting the exosomes of breast cancer cells (Figure 4B). After the aptamers bound to EpCAM and HER2, a toehold nanostructure could be formed under the action of the ligating sequence. Then, the toehold-activated hybrid chain reaction (HCR) was initiated by H1 and H2 hairpin probes, and the EVs were captured by CD36 aptamer to form microbeads. Finally, it was enriched by thermal electrophoresis to amplify the output signal. In the cell experiment, they used three kinds of breast cancer cells (BT-474: HER2, EpCAM expressed; MCF-7: HER2-negative, EpCAM-positive: UACC-812: HER2-positive, EpCAM-negative), and a normal breast epithelial cell (MCF-10: HER2, EpCAM-negative). After detection, this method was found to have a superior diagnostic effect on the BT-474 cell type, and the same confirmation was obtained in clinical samples. The detection limit could be reduced to 2.8 × 10^2^ μL^−1^, which is three orders of magnitude lower than 4.6 × 10^5^ μL^−1^ by flow cytometry [98].

Thermal electrophoresis, with proper aptamer, can already replace some traditional exosome detection methods. By using a new aptamer, which could be less affected by glycosylation than antibody and bind to PD-L1 more effectively, a homogeneous, low-volume, and sensitive extracellular PD-L1 (HOLMES-ExoPD-L1) quantitative method has been developed (Figure 4C). In the thermal electrophoresis stage, Huang et al. focused the infrared laser into the capillary, creating a micron-scale temperature gradient, resulting in the depletion of solvated molecules in the hot spot. In this environment, the thermophoretic depletion of PD-L1 aptamer was significantly faster than that of the exosome–aptamer complex. Under the excitation of the laser, the EVs–aptamer complex will produce stronger fluorescence, while EVs without PD-L1 protein will lead to the rapid depletion of aptamer and produce weaker fluorescence. According to this principle, the diagnosis of adenocarcinoma and the prediction of immunotherapy response can be realized. Subsequent cell experiments and clinical data also support the application prospect of this method in all kinds of adenocarcinomas. PD-L1 exosome can be successfully detected in an environment where its concentration is less than 200 pg/mL, and these samples are mistaken for “PD-L1-negative” in the traditional ELISA method [99].

The greatest value of thermal electrophoresis lies in its amplification of aptamer fluorescence signals, thus further reducing the detection limit [100]. The enrichment of EVs–aptamer complexes by thermal electrophoresis can make its detection effect much higher than traditional techniques such as ELISA and flow cytometry. However, at the same time, it also has high requirements for micron/nanotechnology, which may hinder its wide application in clinical tumor diagnosis.

##### Nanometer Material

**Figure 5:**
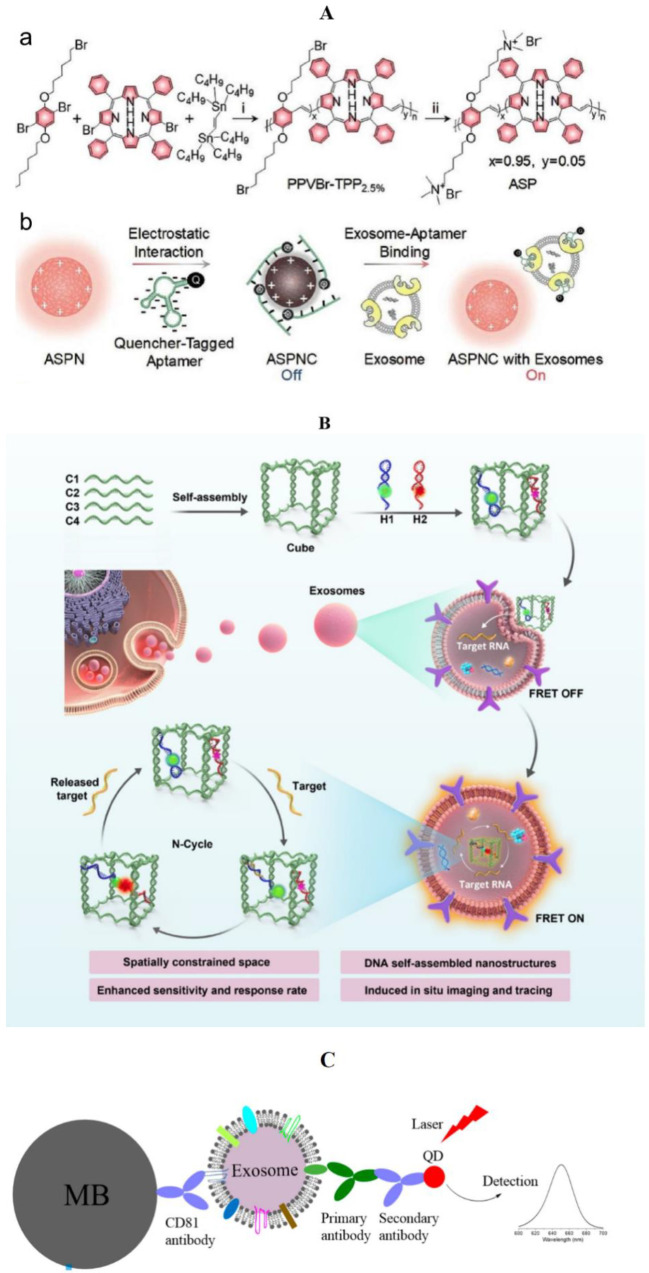
Detection of exosomes in breast cancer by the fluorescence method (nanometer materials). (**A**) Design and sensing mechanism of ASPNC [103]: (a) synthetic route of ASP. (b) Illustration of the formation of ASPNC and the afterglow detection of exosomes; (**B**) schematic Illustration of the assembly process and operational mechanism of DDCA [104]; (**C**) schematic of the QD-based EXO assay [108].

Besides aptamer, nanomaterials are also used in the fluorimetric detection of breast cancer exosomes [101]. In the general fluorescence method, because the exosome separation technique is not mature enough, some biomolecules are often left in samples [102]. These molecules react with fluorescent probes and then produce a background fluorescence signal, which affects detection sensitivity. Due to this, an additional washing operation is required before each detection. In order to solve this problem, Lyu et al. proposed a method of exosome detection based on near-infrared afterglow semiconducting nano-polycomplexes (Figure 5A). They first found that the SPNs of polyphenylacetylene (PPV) could emit a long-life afterglow even after turning off the light source. Based on this property, Lyu et al. synthesized an afterglow semiconductor polyelectrolyte (ASP), and constructed an afterglow semiconductor polyelectrolyte nanocomposite (ASPNC). As a near-infrared photosensitizer, tetraphenylporphyrin (TPP) was introduced to amplify the afterglow signal. By electrostatic attraction, the quaternary ammonium group attached to the complex can form nanocomposites with aptamers labeled by the BHQ-2 quenchant. In the normal state, the fluorescence and afterglow of ASPNC are quenched and leave no signal. When the aptamer binds to the ligand, ASPNC produces fluorescence under light, but its sensitivity decreases due to the interference of biomolecules in the background. After removing the light source, the background signal disappears, while the ASPNC can still produce a long-life afterglow. By detecting this afterglow, background interference can be eliminated and detection sensitivity can be improved. Through the experiment, the levels of HER2 and EpCAM in the exosomes of MCF-7 breast cancer cells were the highest, which is consistent with the biomarkers of breast cancer reported in papers, thus proving the application value of ASPNC [103].

Through DNA nanotechnology, Chen et al. designed a method for the in situ detection of breast cancer exosome (Figure 5B). It does not require any additional amplification technology, and can achieve rapid, sensitive exosome detection. This structure is called double-accelerated DNA cascade amplifier nanostructure (DDCA). DDCA consists of a DNA nano-cube and two hairpin DNA (H1 and H2), in which the sequence of H1 and the target miRNA (miR-21) are complementary. Two fluorescent groups Cy3 and Cy5 were used to modify H1 and H2 respectively. Through base complementary pairing, H1 and H2 were located in a specific position of the DNA nano-cube. When the target exosome was present in the environment, miR-21 and H1 complemented each other to form a H1-miRNA intermediate, which stimulated Cy3 to emit green signal. Then, the H1-miRNA intermediate hybridized with H2 to form a H1–H2 duplex. This duplex made Cy3 and Cy5 close to each other, realizing the "fluorescence resonance energy transfer" (FRET), stimulated Cy5 to emit red fluorescence, and released the target miRNA back to the environment. In this method, the DNA nano-cube can limit the reagent to a compact space with high local concentration, which is called "space limiting effect". At the same time, target miRNAs are released back into the environment after activating the fluorescence signal, which ensures that the exosome concentration stays at certain level. Therefore, this method is called “double-accelerated”. Through the detection of exosomes extracted from MCF-7 cells and blood samples of clinical patients, Chen et al. proved the sensitivity and reliability of DDCA as a nano-probe for the detection of exosomes, and reduced the detection limit to 9.8 × 10^7^ particles/mL, which is 27 times lower than that of conventional methods. In addition, because of the DNA nanoprobe can quickly penetrate the cell membrane, it has also been proved to be able to track the activity of miR-21 exosomes in living MCF-7 cells, suggesting that the nanoprobe can be used as an effective visualization tool to study the function of exosomes in mediating intercellular communication [104].

Quantum dots (QDs) are nanoscale semiconductors with the ability to dissolve in water and combine with specific biomolecules, providing long-lasting fluorescent labeling [105]. QDs are often coupled with antibodies for the detection of exosomes [106]. Wu et al. linked immunoglobulin G (IgG) and streptavidin to quantum dots and successfully labeled HER2 breast cancer markers on the surface of living cancer cells [107]. In addition, the application of quantum dot exosome detection can be broadened by increasing the types of antibodies. Vinduska’s team reported a method for the detection of double-antibody coupled QDs [108]. They used magnetic beads which target CD81 to capture exosomes, coupling the primary antibody to HER2 and the secondary antibody to quantum dots to achieve exosome detection (Figure 5C). The diagnostic ability of this method was proved in the plasma samples of HER2-positive breast cancer patients. The detection only requires microliters of patient’s plasma without any pre-purification and can be completed within 5 hours.

Compared with ordinary fluorescent substances, nanocomposites have unique properties. They can affect the generation and duration of fluorescence, such as afterglow, so as to eliminate background interference, or replace the amplified system by a space-limiting effect. In a word, nanomaterials also have strong versatility [109]. Nanocomposites and aptamers can respectively improve the sensitivity of exosome detection by eliminating background interference, amplifying and stabilizing fluorescence signals. The combination of these two materials can effectively achieve efficient fluorimetric exosome detection of breast cancer.

#### 3.1.3. Surface Plasmon Resonance

Surface plasmon resonance (SPR) is an optical biosensor method that can track biomolecule interactions on metal surfaces in real time. This method does no harm to biomolecules and does not require any labeling [110]. The principle of SPR is using the activation of the metal interface by incident light to conduct electron resonance. The interaction between molecules causes a slight change in the refractive coefficient of the surface, which can reflect the interaction strength of biomolecules and realize the detection of exosomes. SPR is closely related to nanotechnology. With the in-depth study of nano-metal materials, the cost of SPR biosensors has been greatly reduced, making it become one of the newest exosome detection technologies [111].

**Figure 6:**
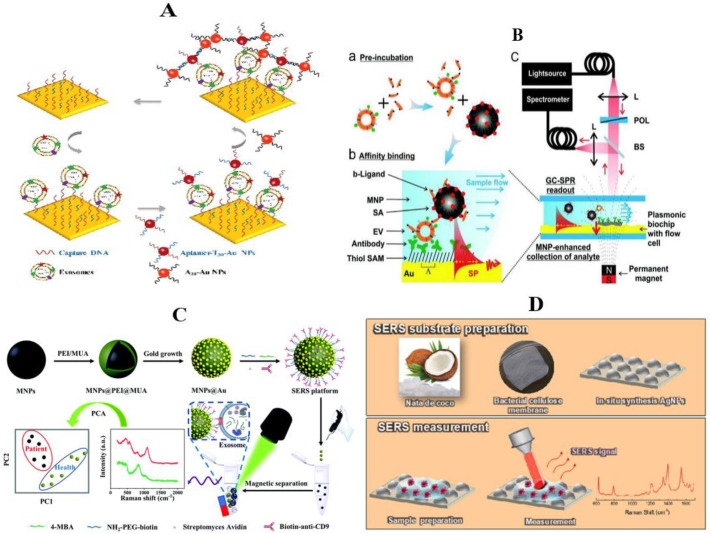
Surface plasmon resonance and surface-enhanced Raman scattering detection of exosomes in breast cancer. (**A**) Dual Au NPs-assisted signal amplification for determination of exosomes [112]; (**B**) schematic of the grating-coupled SPR sensor system and developed assay. SAM, thiol self-assembled monolayer; SP, surface plasmon; L, lens; POL, polarizer; BS, beam splitter; GC-SPR, grating-coupled surface plasmon resonance [113]. (**C**) Schematic diagram of the construction of the magnetic SERS platform and its application for isolation and analysis of breast cancer exosomes [119]; (**D**) schematic representation of the system used in this work. The top panel shows BC membrane production from commercial nata de coco cubes and in situ synthesis of AgNPs into BC for SERS substrates production. The bottom panel shows SERS assay preparation, measurement, and the resulting spectrum [121].

In the study of Wang et al., the synergistic signal amplification effect of Au and Au nanoparticles (NPs) was used to assist SPR technology to achieve sensitive detection of breast cancer exosomes (Figure 6A). First of all, the Au membrane captured the DNA probe and functionalized together, directly detecting the targeted exosomes. Then, they added Au nanoparticles coupled with T30 and aptamers to connect with the detected exosomes. Finally, through the hybridization of complementary sequence T30 and A30, the Au nanoparticles coated with A30 were connected to the aptamer/T30 coupling particles. Through the electronic coupling between Au film and Au nanoparticles, plus the coupling effects of the plasmonic nanostructures, the refraction signal could be amplified. In cell experiments, this SPR sensor can distinguish exosomes from MCF-7 cancer cells and MCF-10A (normal breast) cells, reducing the detection limit to 5 × 10^3^ particles/mL, significantly improving the sensitivity. At the same time, the repeatability and selectivity of the sensor are also confirmed, showing its potential for diagnostic application [112]. The SPR sensor depends on the diffusion-limited binding kinetics of the surface, while the slow diffusion rate of exosomes in the liquid phase limits their enrichment. Reiner and his team improved this limitation by using magnetic nanoparticles (Figure 6B). Before the test began, they concentrated the exosomes on the surface of the sensor in order to improve the mass-transfer efficiency. Meanwhile, they applied a gradient magnetic field to the sensor chip to overcome the limitation of exosome diffusion rate on enrichment, and effectively improve the detection response of the SPR sensor [113].

The SPR method can be used for quantitative analysis without labeling and has the advantages of diversity and real-time interaction [114]. As we can see, the sensor based on SPR is practical in exosome detection. However, at present, SPR technology still has many shortcomings, such as the difficulty to control cost, low stability, and uneven distribution of probes on the metal surface, etc. These deficiencies make it difficult for SPR exosome detection technology to be used in clinical settings.

#### 3.1.4. Surface-Enhanced Raman Spectroscopy

Raman spectroscopy is a label-free technology based on laser inelastic scattering generated by the interaction between the photon and molecular vibration, but the traditional Raman spectroscopy method is limited by a weak signal [115,116]. Therefore, surface-enhanced Raman spectroscopy (SERS) technology came into being. The enhanced Raman scattering method consists of depositing a layer of nano particle shell based on gold or silver onto the surface of exosomes derived from cancer cells and healthy cells to enhance the Raman signal while maintaining the colloidal suspension of a single vesicle [117]. This nano coating can record the SERS spectrum of a single vesicle. Then, through the partial least squares discriminant analysis of the obtained spectra, we can distinguish the exosomes from different sources in the same mixture [118]. Therefore, enhanced Raman scattering can identify single exosomes, which can analyze exosomes more simply and quickly.

Li et al. proposed a magnetic SERS platform to integrate successive breast cancer exosome isolation and Raman signal enhancement into one system (Figure 6C) [119]. They prepared the gold nano-dot-immobilized magnetic nanoparticles (MNPs@Au), and then conjugated them with an antifouling component PEG and a monoclonal antibody (anti-CD9). The built platform could be used to selectively target exosomes with high specific expressions of certain antigens (e.g., CD9). The magnetic SERS platform could completely distinguish exosomes secreted by two different breast cancer cell lines (MCF-7 and MDA-MB-231 cells). Most importantly, this platform can be applied to directly analyze the diluted serum and could also completely screen breast cancer patients from healthy ones.

Because Raman spectroscopy can realize nondestructive analysis without sample preparation, and the required analyte concentration/volume is very low, it is a very ideal method for exosome analysis [120]. However, it requires an active SERS substrate, and the commercial substrate has a high cost and low shelf life. In order to achieve low-cost and green environmental protection, Nuno Ferreira et al. chose composites of commercial nata de coco to produce bacterial nanocellulose and in-situ-synthesized silver nanoparticles as the SERS substrate (Figure 6D) [121]. The lowest detection concentration was 10^-11^ mol. Exosome samples from cell cultures of MCF-10A (nonneoplastic mammary epithelial) and MDA-MB-231 (breast cancer) cells were tested on synthetic substrates. Additionally, it could be used for breast cancer diagnosis.

Raman spectroscopy overcomes the shortcomings of time-consuming operation, requiring professional equipment, and high cost in the identification and classification of genes, proteins, and lipids. It is a simpler and faster exosome detection technology, which can directly obtain the chemical information of exosomes in a nondestructive way. However, washing and magnetic separation after capturing exosomes by Raman scattering lead to the loss and destruction of exosomes, and the additional labeling requirements after capture affect the specificity and sensitivity of exosome detection results. Therefore, more precise exosome detection technology needs to be explored.

#### 3.1.5. Photoacoustic Imaging

Photoacoustic imaging (PAI) is a new biomedical imaging method, which has the characteristics of high spatial/temporal resolution, sufficient imaging depth, and no ionizing radiation [122]. The application of PAI is based on pressure transients generated by the absorption of pulsed or modulated light. Researchers use laser to irradiate the tissue. The optical contrast agent in the tissue absorbs light energy and produces sound-wave transients. The acoustic transducer measures the sound wave at the tissue boundary so as to reconstruct the morphological structure of the part [123]. Endogenous contrast agents include hemoglobin, lipid, water, and melanin, etc., while exogenous contrast agents are mostly small molecular dyes, such as indocyanine green (ICG), methylene blue dye (MBD), and nanoparticles, etc. These small molecules can easily exudate, target cell membrane molecules, and even enter cells, targeting intracellular molecules [124,125]. Therefore, PAI has been widely used in brain functional imaging, thyroid imaging, breast cancer screening, diagnosis of psoriasis and lesions, biopsy and surgical guidance, guidance in the treatment of reproductive and urinary tumors, and imaging of lymph node metastasis [126].

**Figure 7:**
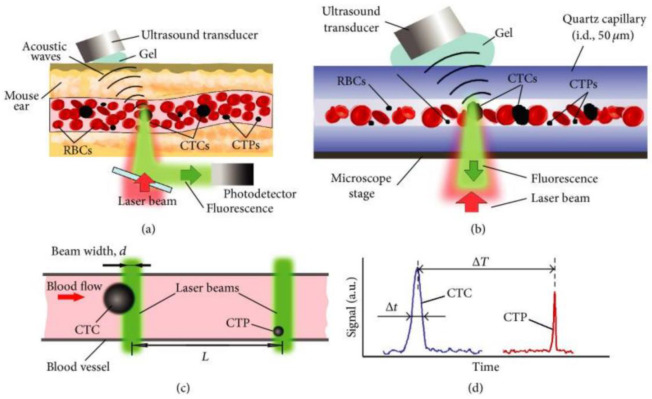
Photoacoustic and fluorescent flow cytometry platform [127]. (**a**) The principle of in vivo detection of CTCs and CTPs using integrated PAFFC schematic. (**b**) The in vitro schematic for detection of CTCs and CTPs in a capillary tube using PAFC. (**c**) Two-beam time-of-flight schematic. (**d**) Signal diagram for CTC and CTP identification in two-beam time-of-flight mode.

Photoacoustic imaging technology is also used in the detection of exosomes of breast cancer. Nolan et al. designed a PAFFC platform by combining photoacoustic imaging with fluorescence flow cytometry to detect Circulating tumor cells (CTCs) and tumor-associated particles (CTPs) in the capillaries of breast cancer mice [127]. The PAFFC uses gold nanorods (GNR) as exogenous contrast agents and targets the surface folate receptor, which is highly expressed in breast cancer cells but is hardly expressed in normal blood cells (Figure 7). GNR was injected intravenously into mice at a dose of 100 μL ∼10^12^ GNRs/mL. After 5 min, the interference signal produced by GNR aggregates disappeared rapidly in the body. Nolan et al. observed the GNR signal in the target breast cancer cells 20–30 minutes later, which confirmed the feasibility of using gold nanorods to detect CTCs and CTPs in breast cancer. Lymph node metastasis is one of the most important factors contributing to the poor prognosis of patients with breast cancer. Piao et al. used PAI to track the lymph node metastasis of breast cancer at the exosome level [128]. They targeted gold nanoparticle (GN) to breast cancer cell-derived exosome (TEX), labeled DC cells, and traced the migration of DC cells to lymph nodes by ultrasound-guided photoacoustic imaging, which provided a theoretical basis for DC immunotherapy.

In short, photoacoustic imaging provides a powerful tool for the early diagnosis of the disease. Because the scattering and absorption of sound waves in tissue are significantly less than those of visible light or near-infrared light, the resolution of PAI is much higher. Compared with the traditional imaging methods, PAI uses the "light in, sound out" technology, with no ionizing radiation and no damage to the patient’s body. At the same time, the contrast agents used by PAI, such as hemoglobin, lipid, water, and so on, exist in various tissues of the human body, which means they have universality in exosome detection. Therefore, it is reasonable to believe that the use of PAI for exosome detection can detect and diagnose breast cancer more accurately, and improve its treatment. In the near future, PAI will have a broader application.

### 3.2. Electrochemical Method

In electrochemical detection, the recognition elements (such as antibodies, aptamers, etc.) are modified on the chemical electrode to specifically bind to the exosomes, and then different electrochemical detection techniques (such as voltammetry, amperometry, impedance method, potential method, etc.) are used to detect the electrode to obtain the concentration and surface protein of the exosomes of the sample [129,130,131]. Electrochemical methods can amplify the signal of reporter molecules by redox and detect low exosome concentrations in small or strongly diluted samples, thus reducing the effects of pollutants (such as protein complexes, lipoproteins, etc.) in the analysis of complex clinical samples (such as plasma). Moreover, because the electrochemical method has the advantages of low cost, good timeliness, high sensitivity, a small number of samples, miniaturization, and intelligence, it is of great significance in the field of biochemical detection and plays an important role in the diagnosis of health care detection (POCT) [132]. The detection of breast cancer exosomes by the electrochemical method has a wide application prospect.

#### 3.2.1. General Material

As a new type of recognition element, nucleic acid aptamer has high affinity and selectivity, and, different from antibodies, aptamer has the advantages of simple preparation, easy modification, and high stability [133]. A synthetic DNA walker is a typical dynamic DNA device, which moves along the DNA track and aids the transduction and amplification of signals with remarkable locomotion and controllability [134]. Structurally, this kind of DNA machine is composed of at least three essential components, including a walker, a track with overhanging branches as footholds, and certain forms of energy input as the driving force. DNA walker has gained tremendous attention in the detection of nucleic acids, proteins, and cells [135,136]. 

**Figure 8:**
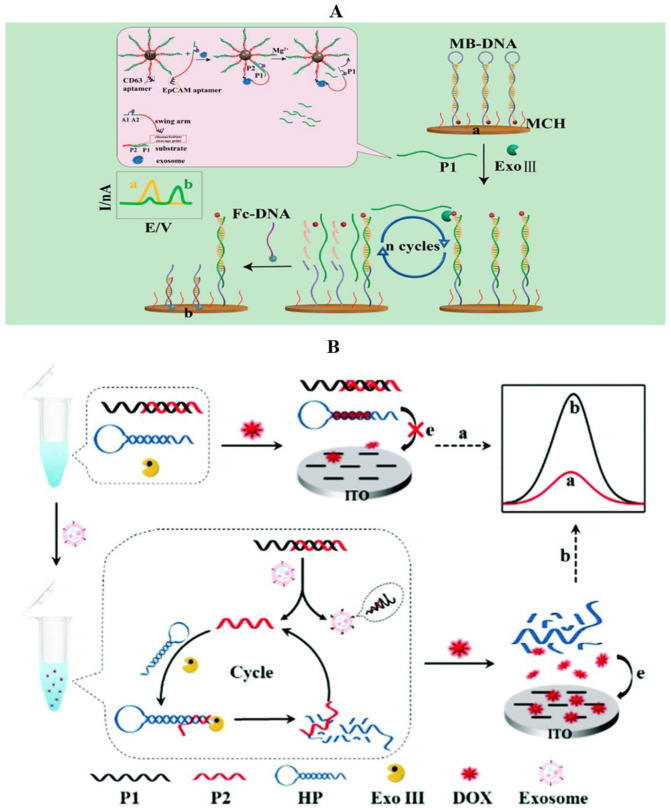
Detection of exosomes in breast cancer by the electrochemical method (general material). (**A**) Schematic illustration for the detection of exosomes through 3D DNA walker amplification and Exo III-assisted electrochemical ratiometric assay [137]; (**B**) principle of the aptamer recognition-trigged label-free homogeneous electrochemical strategy for the ultrasensitive detection of cancer-derived exosomes [142].

Zhao et al. combined nucleic acid aptamers with DNA walkers and developed an ultrasensitive approach for ratiometric electrochemical detection of exosomes by target-triggered three-dimensional DNA walking machine and Exo III-assisted recycling (Figure 8A) [137]. They used CD63 aptamer and EpCAM aptamer to detect the MCF-7 cell-secreted exosome by transforming the detection of exosomes into DNA amplification analysis, using the movements of a three-dimensional DNA walker to amplify the recognition process, and applying the Exonuclease III-assisted electrochemical ratiometric sensor to further amplify the signal to improve the detection sensitivity. Under optimal conditions, the detection limit of this method was 1.3 × 10^4^ particles/mL and it could distinguish the plasma samples from the breast cancer patients and healthy individuals, showing great potential in the clinical assay. Thus, aptamers have great potential in the clinical diagnosis of exosomes.

However, aptamers are not perfect. Because the aptamer probe is usually directly assembled on the electrode surface in conventional electrochemical methods [138], it leads to the spatial hindrance effect, which hinders the effective recognition between the exosomes and aptamer. Additionally, compared with homogeneous solution, the recognition and binding efficiency of aptamer to exosomes on the solution-electrode interface is also relatively low. In addition, in all the reported electrode-based electrochemical methods for exosome determination, the modification of the probe on the electrode surface is sophisticated and time-consuming [139,140,141]. Therefore, the immobilization-free electrochemical protocol has opened up a new way to successfully solve the abovementioned problems. In the homogeneous electrochemical sensing strategy, all the target recognition and signal amplification procedures take place in the homogeneous solution, which greatly improves the detection sensitivity, and the label-free homogeneous electrochemical method also circumvents the expensive signal probe labeling.

By virtue of the anthracycline doxorubicin (DOX) as an excellent electrochemical signal reporter and Exonuclease III (Exo III)-assisted signal amplification technology, an aptamer recognition-trigged label-free homogeneous electrochemical-sensing platform has been reported (Figure 8B) [142]. The nucleic acids used in this detection system typically contain an aptamer probe P1, a trigger DNA probe P2, and a hairpin DNA probe HP. The aptamer probe is used to target CD63 protein, which is enriched in most cancer-derived exosome surfaces. In the absence of cancer-derived exosomes, when DOX is added into the detection system, the electrochemical signal decreases significantly because most of the DOX is be intercalated into P1–P2 and HP probes and there exists electrostatic repulsion of probes toward the indium tin oxide (ITO) electrode, which makes it difficult for the intercalated DOX molecules to reach the electrode surface. In contrast, in the presence of cancer-derived exosomes, the aptamer recognizes and bonded exosomes with high affinity, leading to the release of P2 and triggering the Exo III cleavage process, and then initiating the continual digestion of HP probes. Under these conditions, a great many DOX molecules are free in solution, showing strong diffusivity toward the ITO electrode and resulting in a great current signal. This method was shown to reduce the detection limit for MCF-7 cell-derived exosomes to 1.2 × 10^4^ particles/mL, which is much lower than those of most previous reports. Moreover, the proposed strategy demonstrated excellent selectivity to distinguish the cancer cell-derived exosomes from normal cell-derived exosomes and has been effectively applied to detect the target exosomes spiked in biological samples.

#### 3.2.2. Nanometer Material

Nanomaterials have unique electrical, chemical, and mechanical properties, which are very important in the development of electrochemical sensing. Especially wehen combined with the advantages of different nano-materials, nanocomposites show obvious signal enhancement in electrochemical sensing. Nanoparticles, nanocomposites, and nano-scaffolds can significantly improve the biological characteristics of biomolecules. The combination of nanotechnology and bioelectronic technology can play different specific roles in different electrochemical biosensors [143,144]. Due to the excellent surface area-to-volume ratio of nanomaterials, electrochemical biosensors are more sensitive to external signals; under the synergistic action of biomolecules and nanomaterials, they can further improve the accuracy and accuracy of electrochemical biosensor diagnosis [145].

Metal–organic frameworks (MOFs) are currently the most attractive porous nanomaterial for biosensing applications. As the nanoscaled inorganic-organic hybrids, MOFs not only have the nanoscale enzyme activity described above, but also exhibit several distinct advantages over other existing nanomaterials, including the adjustable structure and surface functionalization for diverse functions, large pore volumes for high loading capabilities, and biodegradable and biocompatible nanoformula for in vivo transportation. Encouraged by biomineralization, multiple biological molecules (e.g., biomacromolecules and cells) are encapsulated into MOFs, thus MOFs are popular for intracellular delivery of nucleic acids and proteins [146,147]. The disassembly of MOFs can be modulated by different endogenous or exogenous stimulus, such as pH, ions, temperature, pressure, redox and magnetic field [148,149,150,151]. 

**Figure 9:**
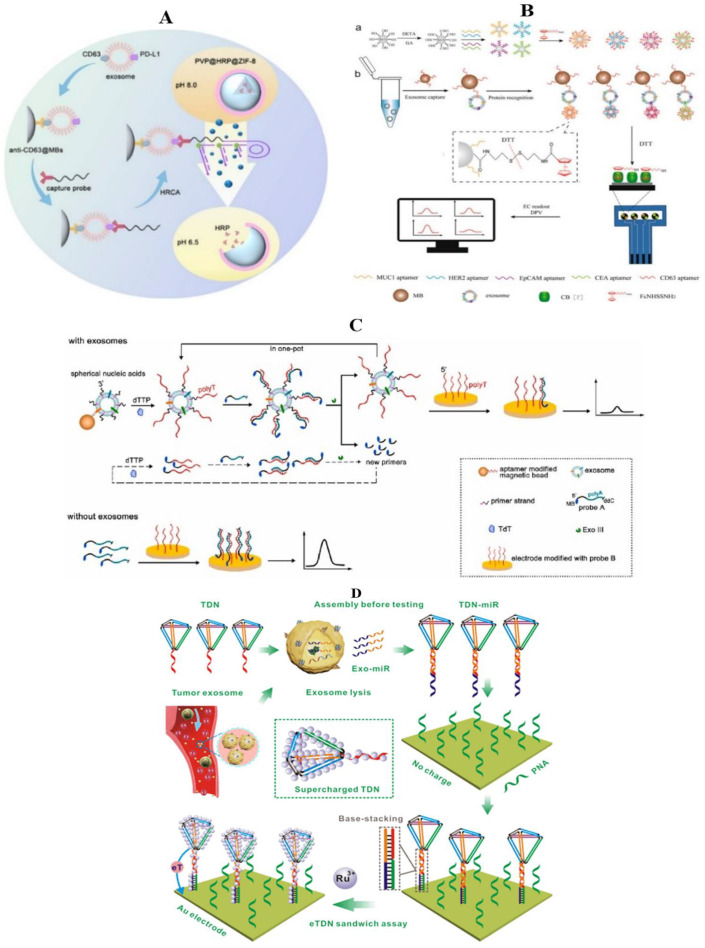
Detection of exosomes in breast cancer by the electrochemical method (nanomaterial). (**A**) Schematic illustration of identification of PD-L1+ exosomes based on HRCA-responsive PVP@HRP@ZIF-8 [152]; (**B**) schematic representation of the magneto-mediated electrochemical sensor for exosomal proteins analysis based on host–guest recognition [154]; (**C**) the principle of the biosensor based on design of spherical nucleic acids and enzyme-mediated triple signal amplification for the electrochemical detection of exosomes. Note: in the presence of exosomes, all reactions are conducted in one-pot, so the magnetic bead is not all depicted in the other steps except the first step [158]; (**D**) schematic diagram of a tetrahedral DNA-nanolabel (TDN) supercharge-based electrochemical (eTDN) sensor for Exo-miR detection [161].

Taking advantage of these characteristics, an electrochemical biosensing method based on DNA amplification-responsive metal–organic frameworks (PVP@HRP@ZIF-8) for accurate identification of programmed death ligand-1 positive (PD-L1) exosomes in breast cancer has been developed (Figure 9A) [152]. HRP is encapsulated in ZIF-8 and then coated with polyvinylpyrrolidone (PVP) to prepare pH-responsive protein-encapsulating ZIF-8 (PVP@HRP@ZIF-8), which is stable at weak alkaline pH and disassembled at acidic pH. PD-L1 exosomes are captured by anti-CD63 functionalized magnetic beads and combined with anti-PD-L1-linked capture probe, and then the surface-attached capture probes are used as primers for in situ hyperbranched rolling circle amplification (HRCA). HRCA lowers the environmental pH and promotes the disassembly of PVP@HRP@ZIF-8, leading to the release of enzymes, leading to amplified electrochemical responses to detect target exosomes. This study is the first to quantify PD-L1 exosomes in the blood samples of breast cancer patients and reveals a potential correlation between the PD-L1 exosome level and the advanced stage and poor prognosis of breast cancer.

The evaluation of exosome-surface proteins plays an important role in the diagnosis and prognosis of tumors [153]. However, due to the lack of adequately accurate and sensitive assay platforms, it is still a significant challenge to analyze the subtle variations of exosomal proteins among different cell subtypes. In the work of An et al., MUC1, HER2, EpCAM, and CEA proteins were employed for the combined detection of breast cancer (Figure 9B) [154]. They developed a magneto-mediated electrochemical sensor based on host–guest recognition for the simultaneous analysis of breast cancer exosomal proteins. They first captured tumor exosomes with CD63 aptamer-modified magnetic beads (MB), then modified silica nanoparticles (SiO_2_ NPs) with MUC1, HER2, EpCAM, and CEA aptamers respectively to identify specific exosomal proteins, and used N-(2-((2-aminoethyl)disulfanyl)ethyl) ferrocene carboxamide (FcNHSSNH_2_) as the signal molecules. The sandwich structure (MB–exosomes–SiO_2_ NPs probe) was separated by a magnet, and N-(2-mercaptoethyl) ferrocene carboxamide (FcNHSH) was released to the supernatant by the addition of reductants (dithiothreitol, DTT) so that the oxidation current signals could be monitored on the graphene oxide-cucurbit 7 (GO-CB [7])-modified screen-printed carbon electrode (SPCE). In this way, four tumor markers on exosomes derived from different breast cancer cells (MCF-7, SK-BR-3, MDA-MB-231, and BT474) could be detected sensitively.

Spherical nucleic acids (SNAs) are a type of nanomaterial with a small spherical core (<100 nm) functionalized with a dense and highly oriented oligonucleotide [155]. Since the nucleic acids shell can improve its binding affinity for complementary oligonucleotides and protein receptors, SNAs have been increasingly applied in biological detection and drug delivery [155,156,157]. Wang et al. proposed a spherical nucleic acids (SNAs)-based cascade signal-amplification strategy for the detection of exosomes with high sensitivity (Figure 9C) [158]. The method targets the high abundance of CD63 and EpCAM251 proteins in the exosomes derived from MCF-7 cells and extends the SNAs anchored to the exosome membranes into polyT strands by terminal deoxynucleotidyl transferase (TDT). The method targets CD63 and EpCAM251 proteins and extends the SNAs anchored on exosome membranes to form a polyT sequence by terminal deoxynucleotidyl transferase (TdT). The modified and blocked signal probe (probe A) is hybridized with polyT strands to form double strands for Exo III-mediated digestion of probe A to produce truncated probe A. Subsequently, the truncated probe A sequence serves as a new primer strand to generate a new polyT strand. Finally, due to the significant decrease in the concentration of probe A, the electrochemical signal is also significantly weakened. This method does not require a complicated nucleic acid sequence design, and also improves the sensitivity of the detection of breast cancer exosomes.

Various nanostructured probes constructed by DNA self-assembly usually feature biocompatibility, programmability, structural order, and addressability, whereas their direct charge contribution to sensitive bioassays remains elusive. The DNA-based nanoassemblies with stable configuration, high yield, and simple fabrication (e.g., single-step assembly) show great potential in charge-based bioassays [159,160]. A typical paradigm is a tetrahedral DNA nanostructure (TDN), which has been widely used in biosensing from heterogeneous surfaces to homogeneous solutions and from extracellular to intracellular settings. Based on these, a supercharged tetrahedral DNA nanolabel-based electrochemical (eTDN) sensor has been reported for the ultrasensitive detection of exosomal microRNAs (Exo-miRs) (Figure 9D) [161]. By using the strategy of “assembly before testing”, the single-step assembled TDN probe hybridizes with the remaining domain in the target Exo-miR, forming a stable sandwich structure together with PNA through the base-stacking effect. TDN-miR carries a significant amount of negative charges and can adsorb a large number of RuHex cations for signal amplification; a short, electroneutral peptide nucleic acid (PNA) probe has also been designed to complement the target Exo-miR and minimize the background signal to achieve high sensitivity (34 aM), high specificity (for single mismatch), and high selectivity (in serum). This ultrasensitive sensor provides a conjugation-free, non-enzymatic Exo-miR detection method in blood and accurately distinguishes breast cancer patients from normal individuals, showing to be a promising tool in the early diagnosis of malignant tumors.

The detection of breast cancer exosomes by the electrochemical method brings a new, efficient, simple, and economical solution to the diagnosis of breast cancer. Although the electrochemical method has the advantages of high sensitivity, simplicity, and rapidness, it is still a major challenge to achieve large-scale clinical applications and strict requirements for experimental conditions in complex testing samples. At present, the diagnostic devices based on electrochemical biosensors used in breast cancer exosome detection still need to be further studied and developed. 

### 3.3. Electrochemiluminescence Method

Electrochemiluminescence (ECL) is a hot technology in the field of analysis and detection, which is a special chemiluminescence reaction initiated by electrochemistry on the surface of the electrode. It couples the dual model of electrochemistry and spectrum, and combines the advantages of electrochemical and chemiluminescence biosensors, such as low background signal, high sensitivity, good controllability, wide detection range, low cost, having a simple optical device, and so on. It is a powerful biological analysis technology [162,163].

**Figure 10:**
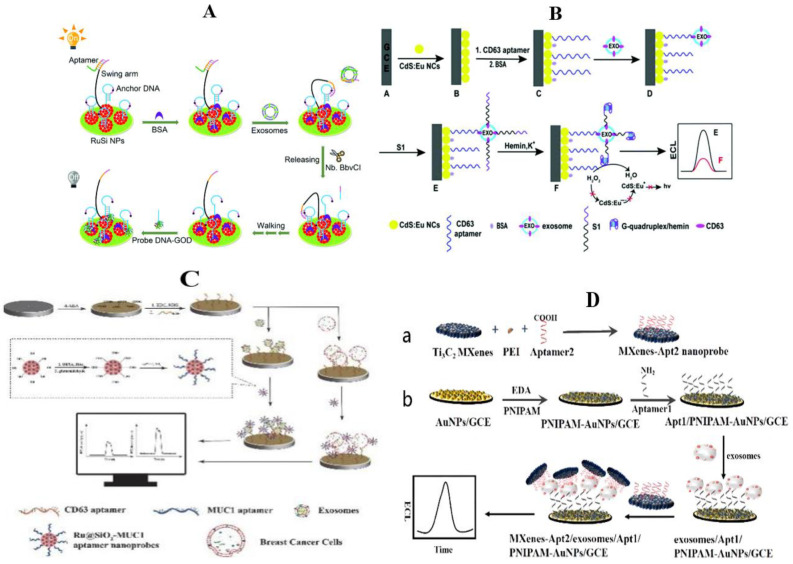
Detection of exosomes in breast cancer by electrochemiluminescence. (**A**) Schematic representation of an aptamer-binding DNA walking machine for ECL detection of tumor exosomes [164]; (**B**) schematic illustration of the ECL aptasensor for exosome detection based on the G-quadruplex/hemin DNAzyme [165]; (**C**) schematic illustration of an ECL aptasensor for the detection of MUC1 protein on breast cancer cells and their derived exosomes [166]; (**D**) the principle of the ECL biosensor for an exosome activity detection signal-amplification strategy [167].

Taking advantage of the high recognition and specificity of CD63 aptamers to the target, Feng et al. reported an aptamer-binding DNA walking machine triggered by the recognition of aptamers to exosomes for sensitive ECL detection of exosomes (Figure 10A) [164]. Their results of using the ECL aptamer sensor to identify exosomes derived from MCF-10A cells are consistent with reference reports.

Nanomaterials have been widely used in ECL and show significant advantages. For example, Qiao et al. reported an ECL aptamer sensor based on G-quadruplex/hemin DNAzymes for the detection of exosomes in MCF-7 cells (Figure 10B) [165]. They used mercaptopropionic acid (MPA)-modified Eu^3+^-doped CdS nanocrystals (MPA-CdS:Eu NCs) and H_2_O_2_ as ECL emitters and co-reactant, respectively, and used CD63 aptamers to recognize and capture exosomes. After the exosomes were captured, they formed a G-quadruplex/hemin DNAzyme, which effectively catalyzed the decomposition of H_2_O_2_, resulting in the decreased ECL signal of MPA-CdS:Eu NCs. At present, the aptasensor has been successfully used to detect exosomes in the serum.

Aptamers and nanomaterials have their own advantages. Aptamer-modified nanomaterials can give full play to their advantages and greatly improve the sensitivity of breast cancer exosome detection. An ECL aptasensor that detects the expression of MUC1 protein in breast cancer cells and their derived exosomes gives full play to the advantages of both. (Figure 10C) [166]. CD63 aptamer has been mobilized on the electrode surface to capture the cells and exosomes and MUC1 aptamer-modified Ru(bpy)_3_^2+^@SiO_2_ nanoparticles (Ru@SiO_2_ NPs) have been used to specifically recognize MUC1 protein on cells and exosomes. This aptasensor has been applied to the determination of MUC1 protein on exosomes in serum samples of patients and normal controls, demonstrating its great potential for clinical cancer surveillance. Additionally, with nucleic acid aptamers and nanomaterials, the aptamer-modified two-dimensional material Ti_3_C_2_ MXenes nanosheets are used as ECL nanoprobes, and the exosomes can be consequently highly efficiently captured onto the electrode surface by an EpCAM protein recognized on the aptamer modified on the electrode surface (Figure 10D) [167]. The ECL nanoprobe has a large specific surface area, good conductivity and catalytic performance, and can recognize exosomes and significantly enhance the ECL signals of luminol. Based on this strategy, the as-prepared ECL biosensor for MCF-7 exosomes exhibited high sensitivity with a lower detection limit of 125 particles/μL, which is 100 times lower than that of the conventional ELISA method, and it has been successfully applied to the detection of MCF-7 exosomes in serum.

The detection of exosomes by ECL analysis provides a powerful tool for the study of exosomal surface protein expression and physiological function. ECL aptamer sensors can be used in the detection of human serum exosomes and have broader application prospects in clinical diagnosis and the prediction of disease prognosis. 

### 3.4. Other Methods

**Figure 11:**
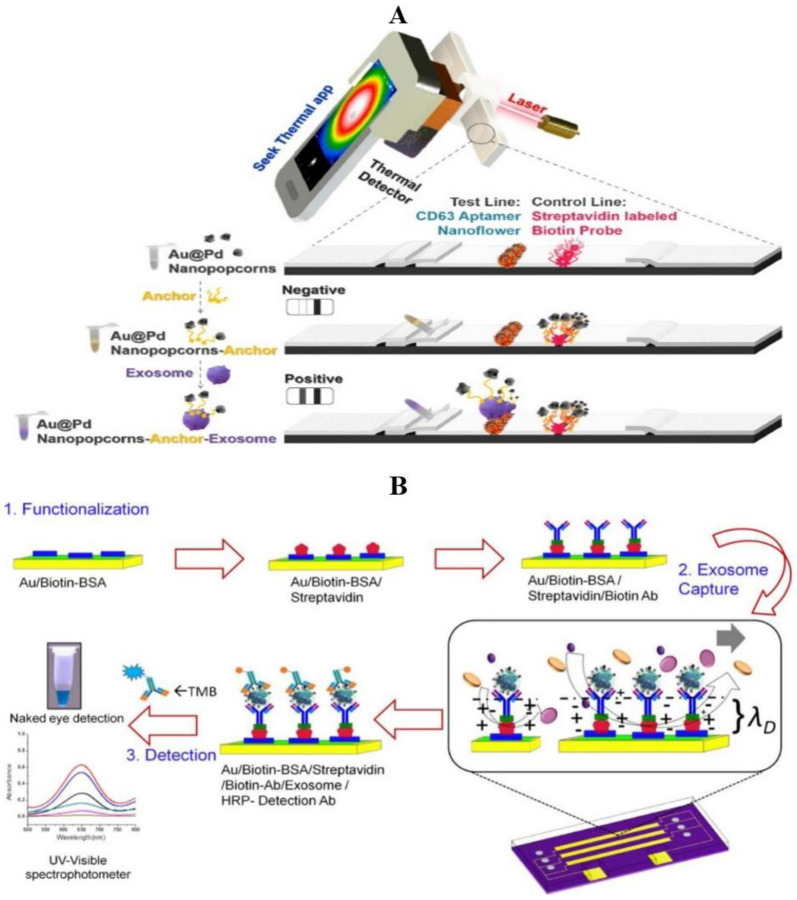
Other methods for the detection of exosomes in breast cancer. (**A**) Schematic illustration of the strategy of integrating an ANAN-LFS with a smartphone-based thermal reader [168]; (**B**) schematic representation of device functionalization, exosome capture, and colorimetric detection of the captured exosomes [169].

In addition to the abovementioned methods, it is also feasible to use thermal signals and other methods to detect exosomes of breast cancer. Cheng et al. developed an Au@Pd nanopopcorn and aptamer nanoflower-assisted lateral-flow strip (ANAN-LFS) with a thermal signal output for the detection of exosomes (Figure 11A) [168]. In this detection strategy, Au@Pd nanopopcorns are used as the signal element in thermal-LFS and aptamer nanoflowers instead of the single-stranded aptamer are used as the capture element on the test line. They also designed a handheld thermal reader based on a smartphone, which makes the strategy a novel, highly sensitive, and portable detection strategy.

Using of the unique ability of an alternating current electrohydrodynamic (ac-EHD)-induced surface nano-shearing, Vaidyanathan et al. reported a multiplexed, microfluidic device for the highly specific capture and detection of multiple exosome targets using a tunable, alternating-current electrohydrodynamic (ac-EHD) methodology (Figure 11B) [169]. This method can enhance the detection ability of the equipment and specifically isolate exosomes from samples of breast cancer patients.

### 3.5. Comparison and Summary of Various Methods

Each of the abovementioned methods has its own advantages and disadvantages. The following is a comparison and summary of various breast cancer exosome-detection methods introduced in this paper (Table 2).

## 4. Summary and Prospects

Fluid biopsy refers to the non-invasive extraction of patients’ body fluids (blood, urine, saliva, milk, etc.) and the analysis of tumor-tissue-derived biomarkers, such as circulating tumor cells, circulating tumor DNA, and exosomes. Compared with the traditional imaging and pathological diagnosis, liquid biopsy has the advantages of being non-invasive, repeatable, and having a timely effect. In recent years, liquid biopsy has shown a sudden increase in popularity, and its clinical applications are becoming more and more extensive, ranging from the detection of early breast cancer to advanced breast cancer, from cancer diagnosis, prognosis prediction, and recurrence risk assessment to drug selection and drug-resistance monitoring. It is not only an effective supplement to histological detection, but also surpasses it in some aspects, and contributes to the accurate diagnosis and treatment of breast cancer.

The exosomes derived from breast cancer cells have significant cell specificity. With the deepening of the study of exosomes, the structural characteristics, basic components, biogenesis, morphological characteristics, and intercellular function of exosomes is becoming clearer. Additionally, through the analysis of exosomes, more and more tumor-related biomarkers have been found, such as HER2, EpCAM, MUC1, CA125, and so on. For breast cancer, many tumor markers such as ER, PR, HER2, MCF-7, CA15-3, and so on can be used as special markers of breast cancer. Based on the newly discovered exosome-labeled proteins, we realized the recognition and detection of exosomes from different sources and enriched the efficient separation and analysis methods of exosomes. We also recognize that exosomes, as an important part of breast cancer fluid biopsy, have their special advantages and disadvantages. Proteins, DNA, miRNA, and other biomolecules in exosome vesicles are blocked by lipid bilayer structures, which are not easily degradable and have good stability. At the same time, drug carriers can be constructed as a means of targeted therapy, but it is difficult to guarantee the purity and biological activity of exosomes.

This article reviewed the latest research progress in the exosome detection of breast cancer. In this paper, optical, electrochemical, and electrochemiluminescence methods were reviewed, and various novel and effective methods for the detection and analysis of exosomes were introduced. The detection of breast cancer-derived exosomes has broad clinical application prospects. It can be used to develop non-invasive diagnostic methods, help to monitor disease progress, efficacy, and drug-resistance mechanisms, and help to judge tumor status. The exosome detection methods reviewed in this paper have the advantages of high sensitivity, simple operation, fast detection speed, and low detection limit. Most of these breast cancer exosome detection methods have been tested in clinical trials, but there are still many difficulties and challenges to achieve a wide range of clinical applications. The traditional separation and detection methods are cumbersome, requiring intensive equipment and workflow. Moreover, once the exosomes are separated, the validation of the exosomes lacks standardization, and the cost is high, which increases the difficulty of applying exosomes to clinical diagnosis and treatment. In fact, exocytosis of exosomes is affected by pH, temperature, hypoxia microenvironment, survival pressure, and other conditions. Therefore, different separation and storage conditions can affect the quality and quantity of exosomes, and different detection methods can also affect the sensitivity and specificity of detection results. We expect that researchers can develop a simple, feasible, efficient, and economic method that can ensure the quality and quantity of exosomes simultaneously. In addition, most exosome markers identified at present are nucleic acid molecules, especially non-coding RNAs such as miRNA. More systematic and high-throughput identification systems need to be developed, more post-translation modified phosphorylated/acetylated/ubiquitinated protein-related biomarkers need to be discovered, and the formation and action mechanisms of exosomes and their contents need to be further studied to guide the development and selection of tumor specific biomarkers. In a word, the current research progress still cannot meet the actual clinical needs of early diagnosis and treatment of breast cancer. Additionally, it is still necessary to further explore the basic biological knowledge of the biogenesis, secretion, target cell uptake, and function of exosomes.

In the future, researchers should focus on developing simple, rapid, and standardized methods for the extraction and detection of exosomes for clinical use. At the same time, we should also enrich molecular mechanism research of breast cancer secreting exosomes and exosomes regulating the occurrence and development of breast cancer, and conduct large-scale validation research on the biomarkers of breast cancer exosomes, so as to predict the existence and biological characteristics of the tumor (such as drug sensitivity, heterogeneity, invasiveness) through the detection of the exosomes, thereby guiding the selection of clinical treatment plans. Therefore, patients will be afforded early diagnosis, effective treatment, and good prognosis of breast cancer through non-invasive liquid biopsy of the exosomes.

## Figures and Tables

**Table 1:** Summary of common methods for exosomes separation.

Method	Principle	Advantages	Shortcoming
Differential centrifugation	According to the difference in density, size and shape, the separation is completed by the cooperation of low-speed centrifugation and high-speed centrifugation.	Easy operation, simple technology, large sample capacity, no additional chemicals, fewer consumables, and good reproducibility	Time consuming, low purity, small quantity, easy contamination [39,40], and may destroy the exosomes’ structure [41].
Density gradient centrifugation	According to the difference in density, size, and shape, the separation is completed by ultracentrifugation in a certain concentration gradient medium.	Compared with ultracentrifugation, the purity is higher, the yield is higher, and no additional chemicals are needed.	High cost, complex technology, tedious operation, time-consuming centrifugation, and loss of a large number of samples [42], which means it is not suitable for large-scale use.
Ultrafiltration method	According to the molecular size or molecular weight, ultrafiltration membranes with different interception relative molecular weights are used to complete the separation.	Suitable for large volume samples, unlimited on sample size, simple technology, less time-consuming, multiple samples can be processed at the same time, and the biological activity of exosomes is not affected [43,44].	Short service life, sample loss leads to reduced output, easy contamination by non-exosome protein, easy deformation of vesicles, and the filter pore is easily clogged.
Size sieving method	According to the molecular size or molecular weight, the separation is completed by a porous gel column.	Compared with ultrafiltration, it takes less time, protects exosome structure, and prevents exosome aggregation.	limited sample volume by column volume, easy contamination by particles of similar size, easy dilution and low yield.
Immune affinity capture method	Separation is completed based on the specific binding between antibodies and exosome membrane proteins, including enzyme-linked immunosorbent assay (ELISA) and immunomagnetic bead adsorption.	High purity and specificity. Most of the target proteins are CD63, CD9, and CD81, which are commonly found on the surface of exosomes [42,45].	High cost, limited output, time-consuming, low extraction efficiency, the antibody can be blocked, the steps of special antibody membrane are cumbersome
Polymer precipitation method	Using hydrophilic polymers or other chemical reagents to change the dispersion or water solubility of the exosomes to precipitate the exosomes.	Easy operation and no need for special instruments.	Low purity, Easy mixing with impurities, need to remove impurities.
Microfluidic method	Controls the fluid behavior in the microchannel to accurately control the droplet shape and particle size.	Light equipment, low sample size, low cost, simple operation, high purity, integration of separation and analysis, process automation [46].	Lack of standard and large-scale testing, low output, special design is needed, high cost.
Artificial antibody method	Based on the mutual recognition between artificial antibody and exosome, such as aptamer technique [47] and molecular imprinting technique [48], exosome separation is completed.	Easy to prepare, economical and applicable, suitable for large-scale use, and universal [49].	Highly professional technology and the kinds of ligands that need to be developed.

**Table 2:** Comparison and summary of various methods for the detection of exosomes.

Type of Biosensor	Exosome Source	Exosomal Biomarker	Bio- Receptor	Probe	Amplification Method	Limit of Detection	Refs.
Colorimetric biosensors	MCF-7 cells, patient’s serum	CD63	aptamer	g-C_3_N_4_NSs nanozyme- H_2_O_2_–TMB	NA	NA	[73]
	patient’s serum	CD63	aptamer	s–SWCNT nanozyme- H_2_O_2_–TMB	NA	5.2 × 10^5^ particles/μL	[74]
	patient’s serum	CD63	aptamer	CuCo_2_O_4_ nanorods	NA	4.5 × 10^3^ particles/μL	[75]
	patient’s serum	CD63	aptamer	Au-NRs	hybridization chain reaction (HCR)	1.6 × 10^2^ particles/μL (spectroscopy) 9 × 10^3^ particles/μL (naked eyes)	[81]
	patient’s serum	CD63	aptamer	Au-NBP@ MnO_2_-NSs	competitive reaction between exosomes, inducing placeholder chains	1.35 × 10^2^ particles/μL	[85]
	MCF-7 cells	CD63	aptamer	Fe–MOF	NA	5.2 × 10^4^ particles/μL	[79]
	MCF-7 cells, patient’s serum	CD63	aptamer	HRP–H_2_O_2_–PDA	NA	7.7 × 10^3^ particles/μL	[57]
	MCF-7 cells	CD63	antibody	HRP-PDA (paper based)	NA	4.46 × 10^3^ particles/μL	[62]
Fluorescent biosensors	MCF-7 cells	MUC1	aptamer	Tamra–Dabcyl	NA	4.2 × 10^4^ particles/μL	[87]
	MCF-7 cells, patient’s serum	CD63 cholesterol	aptamer	Connector–Backbone (proximity ligation)	rolling circle amplification (RCA)	NA	[88] [89]
	MCF-7 cells	HER2 EpCAM	aptamer	ASPNC, BHQ-2	TPP amplify the afterglow signal	NA	[103]
	BT-474 cells UACC-812 cells MCF-7 cells patient’s serum	HER2 EpCAM CD36	aptamer	GelRed H1-H2 toehold nanostructure	hybridization chain reaction (HCR)	2.8 × 10^2^ particles/μL	[98]
	plasma	CA15-3 CA125 CEA HER2 EGFR PSMA EpCAM VEGF	aptamer	Cy-5	localized laser heating for thermophoretic accumulation	3.8 × 10^4^ particles/μL	[97]
Fluorescent biosensors	MCF-7 cells plasma	PD-L1	aptamer	infrared laser	NA	<200 pg/mL	[99]
	MCF-7 cells patient’s serum	miR-21	Fusion protein	Vir-FVs	NA	NA	[92]
	SK-BR-3 cells	HER2	IgG Streptavidin	Quantum dots	NA	NA	[107]
	patient’s plasma	CD81 HER2	antibody	Quantum dots	NA	NA	[108]
Surface plasmon Resonance (SPR) biosensors	MCF-7 cells	CD63	aptamer	Au NPs	electronic coupling plasmonic nanostructures	5 × 10^3^ exosomes/mL	[112]
Surface- Enhanced Raman Scattering (SERS) biosensors	MCF-7 MDA-MB-231 patient’s serum	CD9	antibody	MNPs@Au PEG	NA	NA	[119]
	MCF-7 MDA-MB-231	Rhodamine 6G	bacterial cellulose membrane	Ag NPs	NA	10^−11^M	[121]
Photoacoustic Imaging	breast cancer mice	folate	membrane	gold nanorods	NA	NA	[127]
	DC cells	TEX	NA	gold nanoparticles	NA	NA	[128]
Electro- chemical biosensors	MCF-7 cells	CD63	aptamer	DOX, P1, P2, HP	Exo III auxiliary signal technology	1.2 × 10^4^ particles/mL	[142]
	MCF-7 cells	CD63 EPCAM-251	probe DNA	truncated probe A	TDT extends SNAs into PolyT chain, ExoIII-induced catalytic cleavage	3.8 × 10^4^ particles/μL	[158]
	MCF-7 cells plasma	CD63 EpCAM	aptamer	Au, P1, P2, MCH	3D DNA walking machine and Exo III-assisted recycling	1.3 × 10^4^ particles/mL	[137]
	MCF-7 cells	PD-L1	probe DNA	PVP@HRP@ZIF-8	hyperbranched rolling circle amplification	3.4 × 10^4^ particles/mL	[152]
Electro- chemical biosensors	MCF-7 SK-BR-3 MDA-MB-231 BT474	CD63 MUC1 CEA HER2 EpCAM	aptamer	MB, SiO_2_ NPs	FcNHSSNH_2_ as signal molecule, magnetic separation	NA	[154]
	plasma	miR-21	probe DNA	TDN probe	base-stacking effect	34 aM	[161]
Electrochem-iluminescence biosensors	MCF-7 cells patient’s serum	CD63	aptamer	Cy3, Ti_3_C_2_ MXenes nanocomplex	G-quadruplex/ hemin-DNAzyme, catalyze H_2_O_2_ decomposition	7.41 × 10^4^ particles/mL	[165]
	patient’s serum	MUC1, CD63	aptamer	Ru@SiO_2_ NPs	NA	2.73 × 10^−4^ μg/mL	[166]
	MCF-7 cells	EpCAM	aptamer	Ti_3_C_2_ MXenes nanocomplex	Large specific surface area, electrical conductivity and catalytic performance	5 × 10^3^ particles/μL	[167]
Thermal signal biosensors	Breast cancer cells	CD63	aptamer nanoflowers	Au@Pd nanopopcorn ANAN-LFS	rolling circle amplification	1.4 × 10^4^ exosomes/μL	[168]
Ac electrohy- draulic dynamics	patient’s serum	HER2, PSA	antibody	Au/Biotin-BSA/Strep-tavidin/Biotin Ab	NA	2760 exosomes/μL	[169]

## Data Availability

Not applicable.

## Sample Availability

Samples of the compounds are available from the authors.
